# Sinonasal inverted papilloma involving the middle ear and the mastoid

**DOI:** 10.5935/1808-8694.20120044

**Published:** 2015-10-20

**Authors:** Jônatas Lopes Barbosa, Sebastião Diógenes Pinheiro, Marcos Rabelo de Freitas, André Alencar Araripe Nunes, Elias Bezerra Leite

**Affiliations:** ^1^ MD by the Federal University of Ceará (Resident Physician at the ENT Service of the Walter Cantdio University Hospital of the Medical School of the Federal University of Ceará); ^2^ PhD in Medicine by the Medical School of the University of São Paulo (Adjunct Professor in the ENT Course of the Medical School of the Federal University of Ceará); ^3^ PhD in Medicine by the Federal University of Ceará (Coordinator of the Medical Residency Program and Assistant Professor of the ENT Service of the Walter Cantdio University Hospital of the Medical School of the Federal University of Ceará); ^4^ Specialist in Otorhinolaryngology by the Brazilian ENT-HNS Associaton (Auxiliary Professor and Head of the ENT Service at the Walter Cantdio University Hospital of the Medical School of the Federal University of Ceará); ^5^ Specialist in Otorhinolaryngology by the Brazilian ENT-HNS Associaton (Assistant Physician in the ENT Service at the Walter Cantdio University Hospital of the Medical School of the Federal University of Ceará)

**Keywords:** ear, middle, papilloma, inverted, temporal bone

## INTRODUCTION

Inverted papillomas (IP) are rare benign neoplasms characterized by recurrence and local aggressiveness. They account for 0.5-4% of nasal tumors^1^, ^2^, ^3^. Some etiologies have been proposed, including allergic rhinitis, chronic inflammation, and smoking. There is a possible association with the human papilloma virus (HPV)^1^. HPV subtypes 6, 11, 16, and 18 are among the most frequently found, the first two being the more prevalent subtypes^4^. Middle ear or mastoid involvement is rare. Only 19 cases have been described in the literature published in English by June of 2010^1^.

## CASE REPORT

F.M.A., male, 46, was seen in August of 2008 and complained of nasal obstruction, rhinorrhea and right mild epistaxis evolving for four months. He had been previously diagnosed with IP, as confirmed by histopathology tests. CT scans of his nose and sinuses revealed an IP staged as T2 according to Krouse's staging system (the tumor involved the nasal cavity, the ethmoid sinus, and the medial portion of the maxillary sinus)^3^. The tumor was endoscopically removed and the patient was then followed on an outpatient regime.

Nine months later, the symptoms came back. Nasal endoscopic examination showed a relapsing tumor and CT scans revealed a then T3 neoplasm (the tumor involved the nasal cavity, the ethmoid sinus, the sphenoid sinus, and the lateral portion of the maxillary sinus)^3^. This time, the resection was done using a combined procedure using the endoscopic approach and midfacial degloving.

The patient had relapsing tumors another three times. The last time, on September of 2010, the tumor was protruding out of both nostrils, the patient had a painful, bulging right maxilla, hypacusis, otalgia, and ipsilateral purulent otorrhea. Examination with an otoscope showed a papillomatous lesion covered by purulent effusion occluding the outer ear canal.

**Figure 1:**
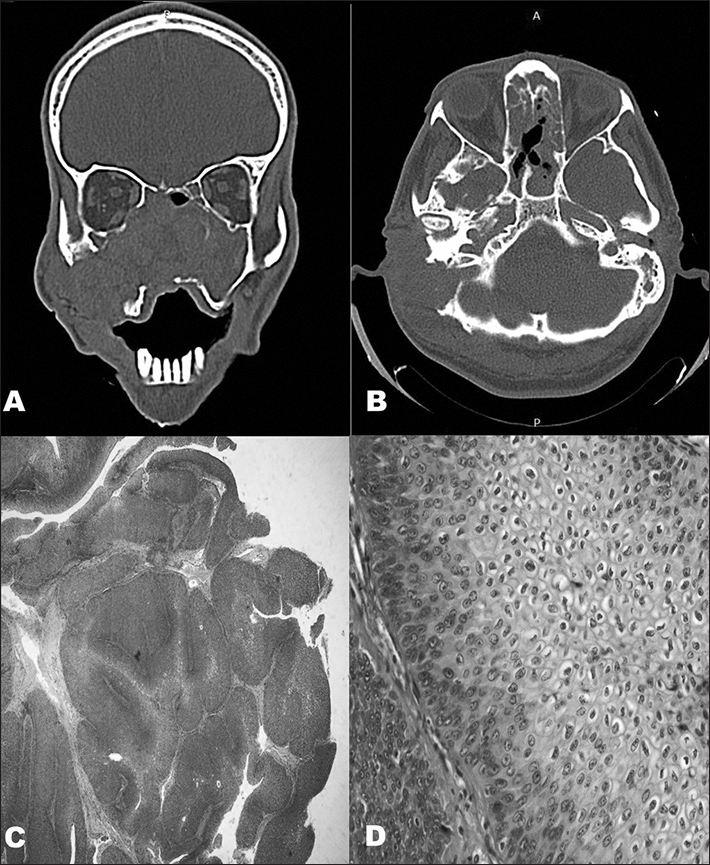
A and B: CT scans of the nose, paranasal sinuses, and mastoid: tumor-like lesion with soft tissue attenuation invading the nasal fossae, the maxillary, ethmoid, and sphenoid sinuses, the rhinopharynx, the right medial orbit wall, and the ipsilateral mastoid, accompanied by massive lysis of the adjacent bone structures. C: Microscope image showing an IP: papillomatous proliferation of the squamous epithelium with endophytic growth pattern (40x - HE). D: Microscope image revealing endophytic projection of the squamous epithelium with preserved cell architecture containing various koilocytes (400x - HE).

CT scans unveiled a tumor occupying the entire nasal fossa and invading the right mastoid, accompanied by intense bone lysis (Figures 1A-B).

The nasal portion of the tumor was removed and, a week later, the patient underwent a radical mastoidectomy. Histopathology confirmed the subject had a non-malignant IP (Figures 1C-1D).

The tumor relapsed once again into the nasal cavity, the right middle ear, and the right mastoid. The patient was referred to radiotherapy and was scheduled to receive a total dose of 5000 cGy divided in 20 sessions. The tumor regressed promptly, but the patient abandoned treatment and died two months later of an unknown cause.

## DISCUSSION

The lateral nasal wall is the preferential primary site for inverted papillomas. Tumors of this type may invade the paranasal sinuses, the orbit, and the anterior portion of the skull base^5^. Middle ear and mastoid involvement is rare^1^, ^2^, ^5^, ^6^. Two theories compete to explain such involvement: one states that it is related to tumors in the sinuses growing toward the eustachian tube, while the other supports the idea that it is due to embryological migration of the Schneiderian mucosa onto the middle ear. Aggression to this ectopic tissue, such as chronic otitis, could serve as stimulus to the appearance of tumors^5^, ^6^.

The signs and signals of nose and sinus disease are non-specific. There may be unilateral nasal obstruction, epistaxis, hyposmia, rhinorrhea, and repetition rhinosinusitis^1^. Middle ear involvement may manifest through hypacusis, aural fullness, and otorrhea. In some cases, papillomatous tumors can be seen in the outer ear canal.

Nose and sinus IP occurs predominantly in males, at a ratio of 4:1^5^, but reviews have shown that in cases involving the temporal bone the ratio changes to 1:1.8. The latter type is just as aggressive as the first, and both can produce intense bone lysis. Rates of transformation into malignant tumors (5-15% in sinonasal IP)^1^, ^2^, ^5^ are higher when there is temporal bone involvement (33%).

Differential diagnosis must consider para-ganglioma, middle ear adenoma, and endolympha-tic sac adenocarcinoma^6^.

The treatment of choice is surgical resection leaving disease-free margins. When there is temporal bone involvement, tympanomastoidectomy is an excellent choice for initial treatment^6^. Another possibility is radical temporal bone resection, but the aggressiveness of this procedure is not justified principally when there is no evidence of malignant disease^1^, ^6^.

Postoperative radiotherapy (RT) is not routinely indicated due to the risk of introducing malignant disease and osteoradionecrosis^1^, ^2^. Nonetheless, RT should be considered in cases in which foci of malignant disease are present in inverted papillomas. RT may also be used when the tumor cannot be completely resected or when it has relapsed multiple times, even in the absence of malignancy. These indication may be extrapolated in cases of temporal bone involvement^1^.

## CLOSING REMARKS

Despite the rarity of inverted papillomas, they must be included in the differential diagnosis of middle ear tumors. The occurrence of otitis media in patients with a history of sinonasal IP should serve as a red flag for the possibility of temporal bone involvement^1^. Surgery is the treatment of preference, but radiotherapy may be considered in select cases.
